# Complement in ischaemia–reperfusion injury and transplantation

**DOI:** 10.1007/s00281-021-00896-3

**Published:** 2021-11-10

**Authors:** Mark C. Howard, Christopher L. Nauser, Conrad A. Farrar, Steven H. Sacks

**Affiliations:** Peter Gorer Department of Immunobiology, School of Immunology & Microbial Sciences, King’s College London, 5thFloor Tower Wing, Guy’s Hospital, Great Maze Pond, London, SE1 9RT UK

**Keywords:** Complement system, Transplantation, Ischaemia reperfusion injury, Antibody-mediated rejection, Innate immunity

## Abstract

Until recently, the only known condition in which complement could mediate transplant injury was the rare occurrence of antibody-mediated rejection, in which the original concept of antibody immunity against the transplant was supported by complementary proteins present in the serum. This has changed within the last two decades because of evidence that the processes of ischaemia–reperfusion injury followed by T cell–mediated rejection are also critically dependent on components generated by the complement system. We now have a clearer understanding of the complement triggers and effectors that mediate injury, and a detailed map of their local sites of production and activation in the kidney. This is providing helpful guidelines as to how these harmful processes that restrict transplant outcomes can be targeted for therapeutic benefit. Here we review some of the recent advances highlighting relevant therapeutic targets.

## Introduction

The complement system is an essential part of the innate immune system, acting both as a first line of defence through pathogen and cell debris removal as well as recruiting components of the adaptive immune system [1]. Recognition of specific patterns on the surface of invasive pathogens or damaged internal cells by pattern recognition molecules (PRMs) activates the complement cascade [2]. Two main pattern recognition pathways are involved: the classical and the lectin complement activation pathways, both of which converge on C3, the pivotal component of complement. In addition, C3 can be cleaved by the alternative pathway, which can start up spontaneously by hydrolysis or can follow the cleavage of C3 to C3b by the classical or lectin pathways. The lectin pathway has a number of PRMs including collectins and ficolins (reviewed in 3, 4), while the classical pathway has only one, C1q [5]. These PRMs initiate the cleavage of early complement components (C4 and C2) to form a C3 convertase, which leads to the cleavage of C3 with subsequent C5 cleavage and generation of the membrane attack complex (MAC), as well as other complement effectors such as C3a and C5a, which mediate inflammation and engage the adaptive immune system [6]. In comparison, the alternative pathway generates a C3 convertase from C3b and factor B (fB), which rapidly increases the amount of cleaved C3 through an amplification loop [7]. While the complement system is an elegant rapid response system and recruiter of the adaptive immune system against invasive organisms, aberrant activation of complement can have a number of detrimental effects on the host. This contributes to inflammatory diseases such as rheumatoid arthritis, age-related macular degeneration and ischaemia–reperfusion injury (IRI) (reviewed in 8). In this review, we will discuss the influence of complement on the pathogenesis of inflammatory disease, particularly in the context of organ transplantation.

## Complement in IRI

Ischaemia–reperfusion injury (IRI) arises in a number of traumatic injuries and diseases, as well as during transplantation. It is characterized by a restriction in blood supply to an organ (ischaemia) followed by the restoration of blood supply and reoxygenation (reperfusion) [9]. While ischaemic damage is related to tissue hypoxia, subsequent reperfusion mediates further tissue damage compounded by an inflammatory response [8]. The role of complement effectors in renal IRI has been extensively described [10, 11]. In addition, more recent studies have identified a primary role for the lectin pathway of complement activation, in particular the contribution of the PRM known as collectin-11 (CL-11) and the potential for blocking its downstream effects [12, 13]. Evidence points to a major fucosylated ligand recognized by CL-11 on hypoxic tissue [13] and has highlighted the feasibility to block CL-11 binding to this ligand by treatment with high concentrations of soluble L-fucose [12]. The alternative pathway has also been implicated because fB^−/−^ mice are resistant to renal IRI [14]. It therefore seems likely that a local tissue ligand recognized by CL-11 leads to complement deposition on the tubules, with further amplification of complement deposition by the alternative pathway. Important to this concept is that complement components (notably CL-11 and C3) produced locally in the kidney at the site of tissue damage have a far greater impact on immune pathology than the corresponding circulating components, produced by the liver and other tissues.

In myocardial IRI, all three complement pathways (classical, lectin and alternative) have been implicated. Myocardial infarction remains a major cause of death in modern societies with the pivotal treatment step being early reperfusion but this can also promote cardiomyocyte death and an increase in the final infarct size [15]. The role of the classical pathway has been investigated through the manipulation of C-reactive protein (CRP), an inflammatory protein that significantly increases at the sites of inflammation during infection. CRP activates C1q, the PRM of the classical pathway [16] and consequently the complement system. Addition of human CRP to rats in a myocardial IRI model increased the resulting infarct size [17] and the addition of a CRP inhibitor resulted in the inverse [18, 19], demonstrating a role for C1q and by inference the classical pathway. The lectin pathway has been also implicated, through observed co-localization of mannose-binding lectin (MBL; a PRM of the lectin pathway) and C3 in rat myocardial IRI models [20]. In addition, the inhibition of MBL led to a reduction of infarct size [21]. Furthermore, loss of MBL associated serum protease-2 (MASP-2) in mice significantly protected against IRI consistent with a role of lectin pathway activation in this process [22]. Therefore, both the lectin and classical pathways of complement activation are considered potential therapeutic targets in this condition (reviewed in 15).

The liver too is susceptible to complement-mediated IRI, unsurprisingly because hepatic synthesis is the major source of circulating complement components such as C3, C4 and MBL [23]. In experimental models of liver IRI involving treatment with C3 inhibitor [24] or C5aR1 inhibitor [25] or induction in C6-deficient rats [26], animals showed decreased post-ischaemic damage (reviewed in 27). Additional data for C6-deficient mice suggest that MAC is essential for tissue damage in the liver, confirmed by further work using CR2–CD59 which binds C8 and C9 to block formation of MAC downstream of C3 and C5 cleavage [27]. Interestingly, the treatment with CD59 not only reduced IRI damage but also increased the rate of hepatocyte regeneration after a partial hepatectomy (in which IRI is part of the surgical procedure) [28]. Furthermore, patients undergoing a partial hepatectomy show increased levels of C3d and C4d, indicating activation of the complement system. In the rat IRI model, IgM and CRP binding to damaged tissue also implicate classical pathway activation via C1q [29].

## Complement links to the adaptive immune response and coagulation

There is extensive crosstalk between the innate and adaptive arms of the immune system. This principally involves an influence on the function of antigen-presenting cells (APCs), such as a dendritic cell (DC), but can also involve direct interaction of innate immune components with B cells and T cells. APCs take up foreign material and present the digested fragments on MHC molecules, which in turn activate specific T cells [30]. On B cells, complement receptor 2 (CR2) is important in cell activation. CR2 is predominantly expressed on follicular dendritic cells (FDCs) and B cells in humans and mice and detects C3-opsonized antigen [31]. As detailed earlier, activation of complement pathways by pattern recognition results in the cleavage of C3 to C3a and C3b. A proportion of the C3b will remain bound to the target antigen surface via protein amine groups. Factor I, in conjunction with complement regulators such as factor H and complement receptor 1 (CR1), converts C3b to C3i and C3d(g). Interaction of C3d-opsonized antigen with CR2 both promotes retention of the target antigen by FDCs in the B-cell areas of lymphoid tissue and augments the stimulation of B cells by the specific antigen [32]. Links between complement and T cell activity have been confirmed in decay-accelerating factor (DAF)–deficient mice. DAF is a regulator of the complement system and mice lacking DAF have increased complement activation and enhanced T cell responses to the reintroduction of antigen [33]. In addition to opsonization by C3b, the complement activation fragment C5a can directly engage C5aR on APC and T cells, to influence T cell immunity. Mice with reduced C5aR activity, induced by inhibitor or targeted deficiency, show a reduction in CD8 + T cell responses to infection [34, 35]. These examples combine to illustrate extensive regulation of the adaptive immune system by complement and complement control proteins [30, 31, 36].

The complement system not only mediates cell and pathogen injury by lysis and recruitment of immune cells, but it also signals to other pathways, including the coagulation pathways. Briefly, two coagulation pathways, the intrinsic and the extrinsic, converge on factor X (FX). The pathways are initiated by tissue factor (TF) exposure (extrinsic) or an activation cascade of factors XII, XI and IX. These ultimately result in the activation of FX to FXa. In association with FVa, FXa then cleaves prothrombin to thrombin which ultimately generates insoluble fibrin and consequently a blood clot [3, 37]. Because MASP-1 resembles thrombin in the crystal structure of its catalytic domain [38], it can cleave fibrinogen amongst other coagulation factors (such as factor VIII) [39]. Both MBL-MASP and L-ficolin-MASP complexes bind to their targets, thus activating the coagulation pathway and resulting in the formation of fibrin blood clots [39, 40]. MASP-2 can cleave prothrombin to generate thrombin which in turn can cleave fibrinogen and factor VIII, thereby forming fibrin clots. Interestingly, the fibrin generated is known to be covalently bound to the same bacterial surfaces that the MBL-MASP2 complex is bound to and can attract phagocytes and/or act as adhesion points for immune system cells. Therefore, this link to the clotting pathway could have direct functional relevance to the innate immune response [41].

## Complement and transplantation

Some of the earliest steps in allograft rejection are mediated by the innate immune system, which plays an additional role enhancing and directing the adaptive immune response to transplantation. There are two peaks of complement activation in transplantation, IRI, as described above, and acute rejection [42]. This complement activation is detrimental in itself as described above but also leads to recruitment of the adaptive immune system, for example by enhancing T-cell activity (reviewed in 43). These varying roles of complement within the transplant process are important in the transplant outcome and will be discussed below.

The first and most direct effect of complement in a transplanted organ is the inflammatory response, which is mediated via generation of biologically active complement fragments. These mediate chemotaxis and neutrophil and macrophage activation. The biological effects depend on the site and trigger of complement activation. For example, in renal IRI, the primary location of complement activation is the tubulo-interstitial compartment of the kidney, which appears to be triggered by locally released CL-11 [14]. In the case of IRI, C3a/C3aR interaction contributes to glomerular and tubular injury [44, 45] and C3a has also been shown to stimulate secondary epithelial cell chemokine production contributing to local inflammation [46]. In native organ studies, from which we can infer information relevant to transplants, it has been shown that C5a is a strong chemoattractant, with C5aR deficiency showing a protective effect in models of ischemia–reperfusion injury [47]. Further evidence of complement in IRI comes from studies using C5 inhibition, as well as lectin blockade to protect from IRI in kidney and cardiac models [22, 47, 48]. Once MAC is formed, cells are induced to produce IL-1α and IL-8, further increasing local tissue inflammation [49].

Complement activation is also critical to the development of adaptive immunity. In an alloimmune response, interactions of antigen-presenting cells (APCs) and T cells are associated with the release of alternative pathway complement components C3, factors B and D, along with C5 [50–52]. There is a corresponding upregulation in the expression of C3aR and C5aR on T cells [53, 54], associated with reduced expression of the complement regulator, DAF. These changes favour local complement activation and enhanced T cell proliferation [53, 55]. Furthermore, studies have identified direct stimulation of B cell priming by opsonized antigens [56, 57], while studies in skin-grafted mice confirm that donor-specific antibody (DSA) production is markedly impaired in the absence of complement activation [58]. Antibody-mediated rejection (ABMR) is due to development of DSAs combined with histologic changes on graft biopsy, with concomitant deterioration in graft function [59]. The role of complement in this setting is well established. ABMR is mediated by the classical pathway, when C1q binds to DSA at the site of endothelial antibody attachment. This mechanism has been described in both acute and chronic transplant injury [60]. Furthermore, it has been shown that complement depletion impairs antibody production via its role in T cell presentation of antigen to B cells [61]. Particularly, there is evidence that DSA interaction with C1q could determine cytotoxic potential of these antibodies and could be used to risk stratify and diagnose ABMR [62]. C5a-C5aR interaction is another important part of ABMR, as shown in mouse allograft studies which have shown that lack of C5aR in both donor and recipient reduced allospecific T cell responses (and therefore improved outcomes). This lack of C5aR also reduced the function of APCs, cellular infiltration and inflammation [63]. Linked to this, recent work has shown that C5 inhibition in highly sensitized mice is protective [64], and importantly, C5 inhibition led to long-term allograft survival despite present DSA [65–68]. Therefore, complement control has both a direct and indirect role to play in the successful outcome of transplants and is an emerging target for therapies.

It should be noted that while IRI and T cell-mediated rejection involve complement activation in the tublointersitial compartment of the kidney, ABMR takes place within the vascular compartment where complement is deposited at the site of antibody binding [69]. Potential therapeutic agents should therefore be appropriately selected for delivery to the appropriate compartment for maximal therapeutic benefit.

## Therapeutics

The effectors of the complement cascade are potential therapeutic targets for the treatment of certain inflammatory conditions. A recent review of these targets illustrates the breadth and indications for such approaches [70]. Here, we focus on recent updates with relevance to kidney transplantation.

The component of choice is partly determined by the severity of the complement-mediated inflammatory condition and the degree to which intervention at this point will interfere with the critical anti-microbial effector functions of the complement system, such as immune complex clearance, opsonization and chemotactic movement of immune cells such as polymorphonuclear neutrophils. Prolonged inhibition of complement activation will lead to individuals becoming susceptible to recurrent infections. To this end, tailored therapeutic strategies aimed to interrupt complement activity in an organ-specific fashion are likely to lead to more favourable outcomes for patients [2].

In the setting of whole organ transplant rejection, there is active research into the application of reagents that can target the complement system, aimed at taming ischaemic damage and subsequent allograft rejection. Eculizumab (anti-C5), marketed by Alexion as Soliris, is licensed as a treatment for paroxysmal nocturnal haemoglobinuria (PNH) [71]. Its application in other pathologies is also currently being investigated, particularly as a method of treating IRI injury associated with kidney transplantation [72]. In a recent review, eculizumab has been shown to protect renal allografts in recipients suffering from post-transplant atypical haemolytic uremic syndrome (aHUS) and ABMR, with positive outcomes over the short term for those patients who are not highly sensitized (reviewed in 73). In a more recent study, with an end point follow-up of 9 weeks post-transplant, in patients subjected to a desensitization protocol, Eculizumab was determined to be effective as a treatment for renal allograft rejection. However, the outcome was heavily dependent upon the method of analysis employed, highlighting the complexity of pathological assessment of patient renal biopsies [74]. In a study focusing on longer term outcomes in paediatric renal transplant recipients, those that were treated with Eculizumab presented with significantly improved early graft function and a significant reduction in chronic glomerulopathy up to 3 years after transplantation. However, an unexpected number of Eculizumab-treated patients had graft rejection following flu-like symptoms suggesting more study is required [72].

Alternative approaches recently employed anti-complement therapeutics have been met with promising results, utilizing a method whereby complement inhibitor can be directly administered to the donor kidney ex vivo prior to transplantation. This approach has the advantage of inhibiting complement activity locally within the kidney, thereby having less impact on the recipient’s systemic pool of complement. Mirococept (derived from human CR1; [75]) was administered at 10 mg per volunteer in a trial (EMPIRIKAL) to assess whether treatment would have a positive impact on the length of delayed graft function (DGF; [76]). The first EMPIRIKAL trial successfully showed the feasibility and safety of the ex vivo delivery of Mirococept to the kidney allograft. It also showed the need for a dose calibration study which was subsequently completed in pig kidney. These dosing studies have demonstrated strong localization to the tubular epithelium and capillaries of the treated kidney up to 80 mg, with minimal release into the recipient [77]. The original EMPIRIKAL trial is now undergoing a revised study protocol for human subjects based on this new higher optimal dose achieved in pigs (EMPIRIKAL-2).

There is scope for therapeutic intervention at the ‘trigger’ stage of complement activation in the setting of inflammatory pathologies. We have recently investigated the principle of sugar blockade to obstruct the carbohydrate recognition site on CL-11 and this therapy inhibits the lectin pathway in a model of renal IRI. Following the intraperitoneal administration of L-fucose in doses sufficient to raise the intrarenal concentration of the soluble sugar, mice were protected against the induction of renal IRI injury as shown by improved renal function when compared to control-treated animals [12]. This is the first demonstration of using a simple sugar to inhibit complement activation in the kidney with beneficial effect, early data in transplanted murine kidney (unpublished) suggesting similar results. This approach is simple and cheap, theoretically leaving anti-microbial functions of the other activation pathways intact.

## Complement inhibitors as promising future therapeutics

A number of complement inhibitors have recently been approved for therapy or are in the final stages of clinical trials and could have an important role in the future treatment of IRI and transplant complications. Firstly, C1 inhibitors (C1-INH) as general serine protease inhibitors, would target the classical and lectin pathways, and although one clinical trial employing a C1-INH has been evaluated as a treatment for ABMR in renal transplant recipients (CINRYZE; Shire ViroPharma Incorporated). The drug was well tolerated, and no demonstrable difference between groups with respect to graft survival was observed, though the C1-INH group trended toward an improvement in renal function. This study was ultimately terminated [83]. However, a separate C1-INH inhibitor (Berinert; CSL Behring) is being tested in a phase 2 study to test the safety and efficacy of this reagent in renal transplant recipients with refractory ABMR [84].

To target complement more generally, work has been undertaken on C3 inhibitors. The non-PEGylated C3 inhibitor compstatin analogue AMY-101 has been developed by Amyndas Pharmaceuticals and they have already announced plans to develop this C3-targeted inhibitor as a treatment option for prevention of organ rejection during kidney transplantation and treatment of patients with end-stage renal disease (ESRD) [85]. Apellis Pharmaceuticals’ complement protein C3 inhibitor Pegcetacoplan has been approved for the treatment of paroxysmal nocturnal hemoglobinuria (PNH) and in a trial has been shown to inhibit both intravascular and extravascular haemolysis, as reflected by increased baseline haemoglobin levels in treated subjects [78]. It is a cyclic peptide and with a small size (43.5 kDa), so application of this technology is pertinent to therapeutic delivery within the kidney, as molecules of this size may penetrate to the corticomedullary junction, the hypoxia-sensitive region of the kidney.

Factor B inhibitors target the alternative pathway as a therapeutic strategy. A novel-specific antisense oligonucleotide (ASO) targeting human complement factor B gene has been developed with the specific aim of reducing circulating levels of this fluid-phase component of the AP, thereby decrease AP activity. The therapeutic, named IONIS-FB-LRX, was evaluated in a phase 1 safety study of healthy volunteers (Clinical trial ref: ACTRN12616000335493). IONIS-FB-LRX reduced plasma factor B levels in a dose-dependent manner in this study. Based on these findings, a phase 2 trial has since been initiated to assess the ability of IONIS-FB-LRX to diminish the growth of age-related macular degeneration (AMD)-associated lesions [79]. This antisense technology could be employed in other inflammatory settings, such as IRI, to temporarily ‘switch off’ the generation of complement intermediates during the acute injury phase. The kidney itself has the capacity to synthesize complement proteins which makes this therapeutic approach an attractive option to pursue further.

Finally, factor D inhibitors would also target the AP. A small-molecule inhibitor of factor D has been developed by Achillion (now part of Alexion/AstraZeneca). This therapeutic is administered orally and has shown promising results in a phase 2 study of PNH, with inhibition of complement-mediated intravascular haemolysis and extravascular haemolysis curtailed significantly [80]. There may be scope for use of small molecules, such as this that could be developed for transplant recipients. The nature of the drug would qualify it for easy penetrance of the renal interstitium and by its design, and its ease of delivery to patients is highly advantageous. These examples indicate an exciting future for the use of complement inhibitors in combating IRI and the detrimental consequences of transplantation.

## Conclusion

**Fig. 1:**
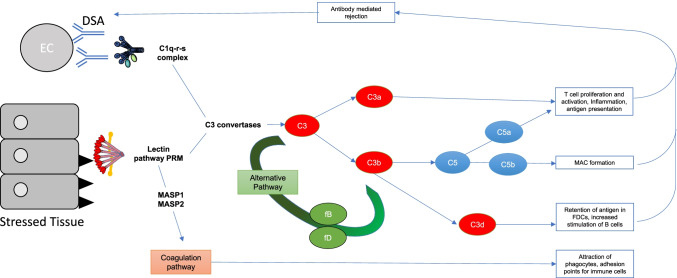
A summary of how complement is activated in IRI and transplantation alongside the downstream consequences. IRI causes stressed epithelial tissue to expose fucosylated ligands (black triangles) which are bound by lectin pathway pattern recognition molecules (PRMs). Antibody-mediated rejection (ABMR) is mediated by donor-specific antibody (DSA) which binds donor endothelial cells (ECs), at sites of inflammation—DSA is then bound by C1q, the PRM of the classical pathway. Both the classical and lectin pathway PRMs activate their respective complement pathways, ultimately producing C3 convertases that cleave C3 to C3a and C3b. C3a, amongst other roles contributes to T cell activation and proliferation. C3b causes activation of the alternative pathway which creates more C3 convertase and therefore a further increase in C3a and C3b. C3b combines with other complement pathway components to create C5 convertases and cleave C5. The resulting products of C5 cleavage are C5a which contributes to inflammation, T cell activation and proliferation and antigen presentation, as well as contributing to ABMR. Another product C5b, alongside complement components C6 to 9 form the terminal complement component, membrane attack complex (MAC). Another downstream product of C3 cleavage, C3d also contributes to retention of antigen in FDCs, and increased stimulation of B cells. Alongside the traditional complement pathway activation, the PRMs of the lectin pathway are also able to crosstalk with the coagulation pathway through the MBL-associated serine proteases (MASPs) 1 and 2 which ultimately results in the attraction of phagocytes, as well as providing adhesion points for immune cells. Epithelial and endothelial injury induced by these processes drives the cycle of complement activation and coagulation feeding back into the pathways described above

The role of complement in transplantation has grown as a field of study in recent years as it has become clear how great an effect it has on the success or failure of the procedure, at least in experimental models. IRI is a key driver of complement activation at the onset of the transplant procedure, as has been demonstrated in a number of organs but is not the whole story, as the crosstalk between the complement system and the greater immune system and the coagulation system also has roles to play (summarized in Fig. 1). This broader perspective of the role of complement in transplantation has led to emerging therapeutic strategies, which we anticipate will take several more years to validate for clinical use.

Unsurprisingly in the context of COVID-19, complement has emerged as a likely contributory factor given the constellation of hyperinflammation, vasculitis and microthrombosis (reviewed in [81]). Furthermore, a recent study showed 46% of patients with COVID-19 had acute kidney injury (AKI) [82]. It remains unclear whether the immune pathology of the AKI is secondary to viral infection of the kidney or is part of the systemic inflammatory response that associates with the acute lung injury. Nonetheless, it is possible that the mechanism of AKI reviewed here may add understanding of how renal involvement is a complication of this condition, to which transplant patients are also vulnerable.

## Data Availability

N/A.

## Code availability

N/A.
