# Correction to: Complement in ischaemia–reperfusion injury and transplantation

**DOI:** 10.1007/s00281-022-00924-w

**Published:** 2022-03-18

**Authors:** Mark C. Howard, Christopher L. Nauser, Conrad A. Farrar, Steven H. Sacks

**Affiliations:** Peter Gorer Department of Immunobiology, School of Immunology & Microbial Sciences, King’s College London, 5thFloor Tower Wing, Guy’s Hospital, Great Maze Pond, London, SE1 9RT UK

**Correction to: Seminars in Immunopathology (2021) 43:789-797**

10.1007/s00281-021-00896-3

The order of Reference 78–85 were incorrect. Here is the correct information:
